# Lasting mantle scars lead to perennial plate tectonics

**DOI:** 10.1038/ncomms11834

**Published:** 2016-06-10

**Authors:** Philip J. Heron, Russell N. Pysklywec, Randell Stephenson

**Affiliations:** 1 Department of Earth Sciences, University of Toronto, 22 Russell St. Toronto, Ontario, Canada M5S 3B1; 2 School of Geosciences, University of Aberdeen, Aberdeen AB24 3UE, Scotland

## Abstract

Mid-ocean ridges, transform faults, subduction and continental collisions form the conventional theory of plate tectonics to explain non-rigid behaviour at plate boundaries. However, the theory does not explain directly the processes involved in intraplate deformation and seismicity. Recently, damage structures in the lithosphere have been linked to the origin of plate tectonics. Despite seismological imaging suggesting that inherited mantle lithosphere heterogeneities are ubiquitous, their plate tectonic role is rarely considered. Here we show that deep lithospheric anomalies can dominate shallow geological features in activating tectonics in plate interiors. In numerical experiments, we found that structures frozen into the mantle lithosphere through plate tectonic processes can behave as quasi-plate boundaries reactivated under far-field compressional forcing. Intraplate locations where proto-lithospheric plates have been scarred by earlier suturing could be regions where latent plate boundaries remain, and where plate tectonics processes are expressed as a ‘perennial' phenomenon.

## Abstract (web summary)

The causes of intraplate deformation remain poorly constrained. Heron *et al*. use numerical models to show that ancient plate tectonic processes produce mantle lithosphere structures that may be reactivated to generate intraplate deformation.

## Graphical abstract

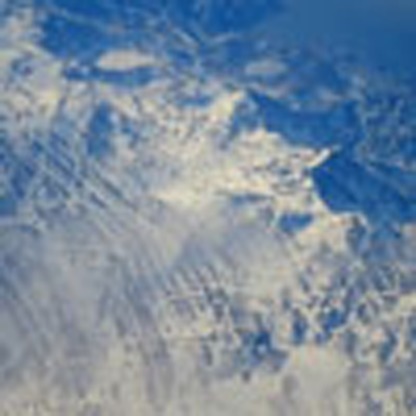

The expanded regions of future (and past) orogenesis may be determined by a ‘perennial plate tectonics' map (Fig. 1), which highlights active and latent plate boundaries and deformation zones. Seismic reflection data from Canada, the Alps, the Pyrenees, the North Sea, and the Skagerrak and Hebrides shelf have identified tectonic structures as scarred lithosphere extending deep into Earth's interior^1^,^2^,^3^ (yellow crosses, Fig. 1). Such seismic reflectors in the mantle lithosphere (ML) have been interpreted to be related to relict subduction zones linked to the closing of oceanic basins (that is, ‘subduction scars' generated at an ancient plate boundary) (for example, refs 4, 5, 6).

ML scars are well established in the geological record (for example, refs 1, 4, 6) but they have not received the same attention as, for example, the reactivation of faults within the Earth's crust^7^ for localizing intraplate deformation. Experiments on rock properties find that deformation generates weak zones that, over time, can be dormant (or be reactivated) depending on how the material strength is affected by changes in ambient stresses. A reduction in grain size is a characteristic of this lithospheric damage^8^, which can be abundant at tectonic margins in the form of ML peridotite mylonites^9^,^10^. As deep seismic reflector anomalies are generally inferred to represent deformation relating to ancient tectonic margin processes (for example, refs 4, 11), these ML scars can be classified as weak zones (that is, a region of reduced grain size at mid-lithospheric depth) via the continuum theory of damage mechanics^8^,^12^.

Here we show the results of thermo-mechanical finite-element experiments with continental parameters to understand further the role of ML heterogeneities by comparing weak zones at different depths and of different geometries. We implemented a strong ML to model the conditions required for a normal continent (for example, (ref. 13)), with a lower crustal rheology that permits crust–mantle interaction (for example, ref. 14). We model pre-existing zones of weakness (for example, (refs 15, 16, 17)) by specifying a region with a low angle of internal friction (to mirror the processes of lithospheric damage (for example, ref. 8)), and simulate inherited structures in the upper crust (UC), lower crust (LC) and ML. We have chosen a two-dimensional (2D) geometry (over three-dimensional) so that the experiments track the evolution of fault zones in the crust–mantle tectonic models to high resolution (1-km vertical resolution in the crust).

## Results

### Analysing the role of ML scars on tectonics

Figure 2a shows 11 differently angled weak zones in both UC and LC, and one ML scar. We then compare convergent (1 cm per year) models after a significant amount of shortening (350–450 km) that feature only UC scars (Fig. 2b), LC scars (Fig. 2c), UC and LC scars (Fig. 3a), and all the available weak zones (Fig. 3b).

The reactivation of crustal scars is time-dependent, with shifting strain patterns occurring once a fault has localized over time^18^. The time-dependence of the UC scars is shown in Fig. 2b by markers A, B and C, with these faults reactivating in alphabetical sequence as strain is accumulated and transferred. The UC fault reactivation produces folding in the UC, but acts to thicken the LC and produce subcrustal subduction within the ML. When only LC scars are present (Fig. 2c), a time-dependent strain pattern change remains (markers D–G) but the thickening of the LC produces widespread folding in the UC.

The combination of both LC and UC scars (Fig. 3a) produces a shifting pattern of strain that appears to be controlled by the lower crustal heterogeneities rather than the shallower features. Timing of crustal fault ruptures (H–K) coordinate with those of LC scars only (Fig. 2c). Furthermore, the combination of LC and UC faults produces a strong decoupling of the LC from the UC, as shown by the high strain rate along the boundary.

The introduction of a ML scar to convergent models featuring 22 remnant crustal features produces a marked focusing of deformation (Fig. 3b). The ML scar supersedes the shallower pre-existing faults to produce ‘pseudo-subduction' (that is, underthrusting of the ML). Lower crustal thickening impinges on the UC to produce folding. Over time, the folding propagates as the convergence continues. However, the deformation produced by the ML scar is localized (despite the numerous crustal features present) with little time-dependence. In fact, the pattern of deformation produced by UC, LC and ML weak scars (Fig. 3b) is almost identical to that of a convergent model featuring a ML scar only (Fig. 3c). Subsequently, the evolution of intraplate deformation follows the later stages of a continent collision (for example, ref. 19).

### Model validation

Additional experiments were performed to test how robust the model set-up is to changes in rheological parameters. Supplementary Fig. 2 shows the strength profiles for the rheology used in Figs 2 and 3 (Supplementary Fig. 2a), as well as for the additional experiments (Supplementary Fig. 2b–d). The models presented in Supplementary Figs 3 and 4 show ML scars to be important in activating tectonics, while the reduction in crust and mantle strength may also determine the dominant factor in tectonic evolution. The models presented in Figs 2 and 3 are the most relevant as we apply a strong LC and ML to simulate relatively stable, strong continental interiors that feature long-lived ML scars. We present additional models here to understand the limits of our study.

### Jelly sandwich or crème brûlée

Supplementary Fig. 3 shows results from using a crust and ML rheology that is not as strong as those experiments presented in Figs 2 and 3. We implement a different configuration of weak zones for this suite of models (Supplementary Fig. 3a) as compared with Fig. 2a, with 12 weak zones in the LC and UC and 2 in the ML. We acknowledge that previous studies have highlighted the possible ‘jelly sandwich' or ‘crème brûlée' strength profiles of the mantle (for example, ref. 13), and present experiments taking this into account in Supplementary Fig. 3. Our jelly sandwich experiments have a weaker LC than shown in Figs 2 and 3 (strength profile as given in Supplementary Fig. 2b). For models just featuring UC scars, we find that the UC dominates tectonic evolution in this jelly sandwich setting (Supplementary Fig. 3b). The time markers A–C show the evolution of strain within the UC.

The introduction of weak zones into the LC and ML (Supplementary Fig. 3c) generates more localized deformation. Although the UC weak zones are reactivated, their influence on regional tectonics and the deformation of the LC is minimal. ML scars again show to be dominant in activating tectonics.

In applying a crème brûlée rheology, we detach the LC and mantle from the UC leading to décollment tectonics (for example, ref. 13). Our results indicate that regions where a crème brûlée rheology is present, perennial plate tectonics may not persist. However, in order for stable continents to be preserved over time, Burov and Watts^13^ found that a jelly sandwich rheology would be required over a crème brûlée rheology. As a result, the crème brûlée' rheology is not applicable to our study of intraplate tectonics.

### LC strength

The strength of the LC and ML is important in activating tectonics (for example, refs 14, 18, 20, 21). To show that our results are not dependent on the rheological parameters, we present a suite of models that use material values that are similar to a previously published paper^22^. In these models we use the weak zone configuration as shown in Supplementary Fig. 3a, and implement flow laws for wet quartz for the UC^23^, wet anorthite for the LC^24^ and dry olivine for the upper mantle^25^ (Supplementary Table 2). These rheological parameters produce strength profiles that are intermediate in the range of published flow laws for crustal and mantle materials (Supplementary Fig. 2d).

Supplementary Fig. 4 shows the material deformation after 270 km of shortening for the rheological parameters given in Supplementary Table 2. The UC weak zones in Supplementary Fig. 4a generate strong folding of the crust. The changing patterns of stress build-up shifts to produce pseudo-subduction of the crust into the ML. The implementation of wet anorthite for the LC generates a layer that is not primed for failure (even with regions of designated weakness). The wet anorthite flow law generates a weak LC that plays no role in the deformation pattern. This is shown with the introduction of UC and LC weak zones (Supplementary Fig. 4b) and the dominance of the upper crustal weak zones in the deformation pattern.

The ML weak zones again dominate the deformation pattern when introduced in Supplementary Fig. 4c. Although the UC weak zones reactivate, the overall tectonic pattern is controlled by the ML scars (with the LC weak zones playing no role). The time-dependent nature of the UC deformation pattern is replaced by the ML scarring's ability to accommodate shortening through sub-crustal subduction. In an experiment featuring only ML scars (Supplementary Fig. 3d), the deformation is localized. However, the pattern of crustal deformation with all weak zones present (Supplementary Fig. 4c) follows closely with that of the simulation with just ML weak zones (Supplementary Fig. 4d), rather than that of UC only (Supplementary Fig. 4a), implying that the deeper scars control tectonic evolution.

### Plate velocity

By increasing the convergence rate of the model from *υ*=1 to 2.5 cm per year, we can show the difference in deformation due to plate forcing. Here we compare simulations that have been shortened by 450 km using the rheological parameters of Supplementary Fig. 2a and Supplementary Table 1 (and the weak zone configuration shown in Fig. 2a). The only major difference that occurs from increasing the convergence velocity (when comparing with Fig. 2) happens for the model featuring UC scars only (Fig. 2b and Supplementary Fig. 5a). At a lower convergence velocity the stress pattern changes over time reactivating faults over time (Fig. 2b and Supplementary Fig. 2b). However, at *υ*=2.5 cm per year the stress pattern does not change and the time-dependence of the model disappears. In the presence of a higher convergence velocity, the result that lower crustal weak scars control deformation over shallower features is highlighted due to this lack of time-dependence with UC scars. The deformation pattern from UC and LC scars (Supplementary Fig. 5c) is more similar to LC tectonics (Supplementary Fig. 5b) than UC tectonics (Supplementary Fig. 5a). Overall, increasing the convergence velocity allows for the same conclusions: deep lithospheric heterogeneities can control deformation over shallower features.

### ML weak zone orientation

Supplementary Fig. 6 give an indication of how the orientation of a ML weak scar would affect the pattern of deformation. In all previous models we applied similar angles to the scars within the ML. Supplementary Fig. 6a gives four different ML scar configurations: ML(i) two scars with angles as previously shown; ML(ii) two steeper ML scars; ML(iii) two vertical ML scars; and ML(iv) two smaller ML scars (of the same size as the lower crustal scars). All models are realized with UC and LC scars as in Supplementary Fig. 3a. Models ML(i), ML(ii) and ML(iv) exhibit very similar deformation patterns despite having different configurations. In a result shown here, we found that if a ML scar becomes small enough, the crustal scars can control deformation.

The vertical nature of the weak scars in model ML(iii) inverses the deformation pattern, which indicates that there appears to be a preferred angle in which the ML can pseudo-subduct. However, the overall result from changing the orientation of the weak scars in the ML is that deeper heterogeneities often controls the pattern of deformation (despite changing the angles of scars).

## Discussion

Our results demonstrate that deformation patterns in continent interiors are influenced by the depth of lithospheric heterogeneities, and that ML scars can guide deformation over other pre-existing features in the UC and LC. The range of deformation patterns exhibited from the models presented here indicates the complex nature of lithospheric heterogeneities and plate tectonics. The activation of dormant lithospheric structures depends on changes in the regional stress regime^18^.

The tectonic history of China provides one reference to understand plate tectonics beyond plate boundaries with regards to our results. Although there are many regions where continents have been accreted by closure of paleo-oceans between micro-plates, China is a unique reference as the far-field convergent stress from the Indian–Eurasian collision is relatively recent and ongoing (Fig. 4a).

The Altyn Tagh Fault (ATF), on the northern margin of the Tibetan Plateau, has a distinct present-day ML heterogeneity linked to a continent–continent suture^26^. The ATF accommodates some of the convergence between the Indian and Eurasian plates^27^, and is characterized by localized deformation that has produced ∼475±70 km of staggered displacement since the mid-Oligocene^26^. Although focal mechanisms of earthquakes close to the ATF show strike–slip motion, compressional processes account for earthquakes to the south^27^, with numerous thrust faults also inhabiting the area (Fig. 4b). Geophysical studies of the ATF show deformation that penetrates the entire crust to link to heterogeneous structures in the ML^27^,^28^,^29^ (Fig. 4c).

Supporting our findings, the ATF can be interpreted as a ML scar originating as a continent–continent collision in the Palaeozoic^30^ that controls intraplate deformation during periods of compression (with the most recent episode starting in the Oligocene by the India–Eurasia collision). A similar process could be considered for the crustal deformation in the presence of ML heterogeneities beneath the Alps, the Laramide, the Pyrenees and the Eurekan orogens^31^,^32^,^33^, as well as deformation in the Tien Shan, the Caucasus and the southeastern Ukraine (for example, refs 34, 35 and references therein). However, geological examples exist where lithospheric folding in relatively strong oceanic lithosphere has been generated due to age-dependent plate tectonic forces. An example in particular being buckling during the recent Eurasian convergence in the northeastern Indian Ocean^36^, results of which are supported by numerical modelling of net plate tectonic forces^37^. The role of plate tectonic forces in concentrating stresses inside plates is a mechanism that may need to be incorporated into this ML scarring method of developing intraplate deformation.

Even though the choice of crust and ML rheology is indicative of a stable continent^13^,^14^, it may not be applicable to all geological settings. Supplementary Fig. 3 shows that continental lithosphere rheology is important in the generation of crustal deformation (through coupling dynamics) in agreement with recent analogue models (for example, refs 21, 38, 39, 40). Our results offer an explanation as to why preserved structures in the continental crust do not always reactivate during periods of compression; the localization of deformation from strong ML heterogeneities can change strain patterns during periods of shortening and dominate over the crustal response. Furthermore, our result that ML dominates crust in controlling tectonic processes is consistent with findings from geological studies (for example, refs 11, 20, 41) and recent physical scaled analogue modelling of heterogeneous lithosphere (for example, refs 21, 38, 39, 40) (although no previous studies have incorporated both crustal and mantle heterogeneities as done here).

ML scars related to oceanic subduction have been the most documented in the geological record (for example, refs 2, 3, 4, 5, 6). However, processes that do not feature oceanic subduction may also account for seismically inferred heterogeneities. Rayleigh–Taylor instability, lithospheric underplating, lithospheric delamination and lithospheric subduction are all related to continental collisions (that is, suture zone tectonics) and may produce lasting (seismically visible) ML structures^11^,^42^. An increase in the number of high-resolution imaging studies of the upper ML (at sites of previous continental collisions in particular) would be able to test this extended theory through the discovery of further ML heterogeneities.

**Figure 1:**
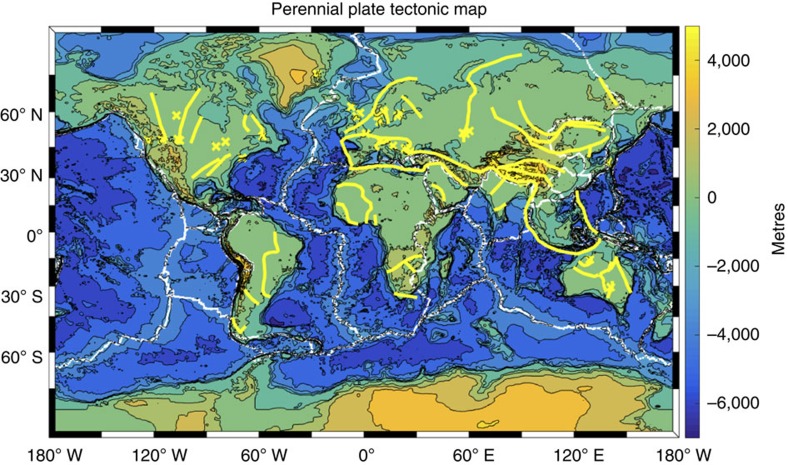
A perennial plate tectonic map. Present-day plate boundaries with major paleo-suture zones and regions of ML scars. Yellow lines are regions of proposed ancient suture zones as compiled from Burke *et al*.^46^ and Hoffman *et al*.^47^. Yellow crosses indicate seismic surveys where mantle reflections have been reported, with locations here given by a previous compilation of mantle heterogeneities^1^ and two recent studies^6^,^48^. Present-day plate boundaries are white lines as compiled from Bird^49^.

**Figure 2:**
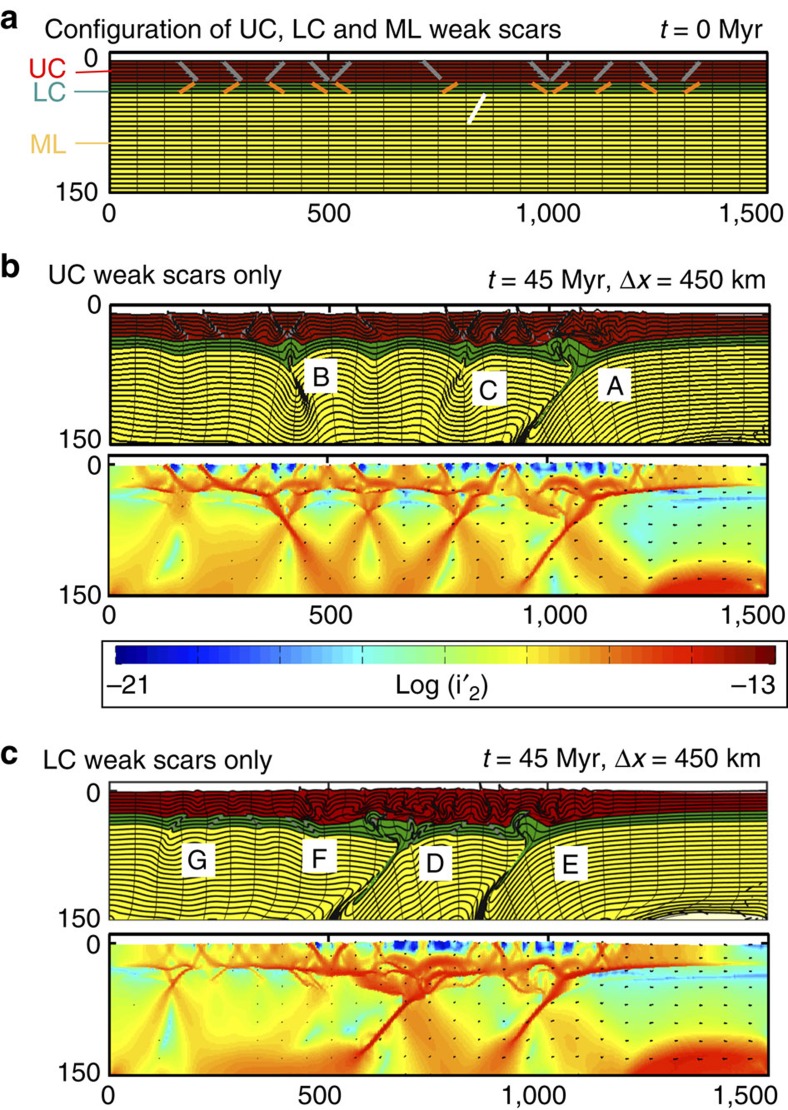
Model set-up and results with UC and LC weak zones. (**a**) Model set-up (red: UC (wet quartzite); green: LC (Maryland diabase); yellow: ML (dry olivine)). The top 150 km of the 600-km deep model is shown at the initial condition (*t*=0 Myr) with the configuration of UC, LC and ML weak scars. The full width of the model is shown. Continental convergence is incorporated by introducing new lithosphere at the right boundary of box with velocity *υ*=1 cm per year. (**b**). Material deformation (top) and visualization of the second invariant of the deviatoric strain rate tensor (bottom) after 450 km of shortening for only UC weak scars. (**c**) As **b**, but for LC weak scars only. The alphabetical markers (A–G) show the progression of the deformation throughout the full model simulation and track the evolution of tectonics.

**Figure 3:**
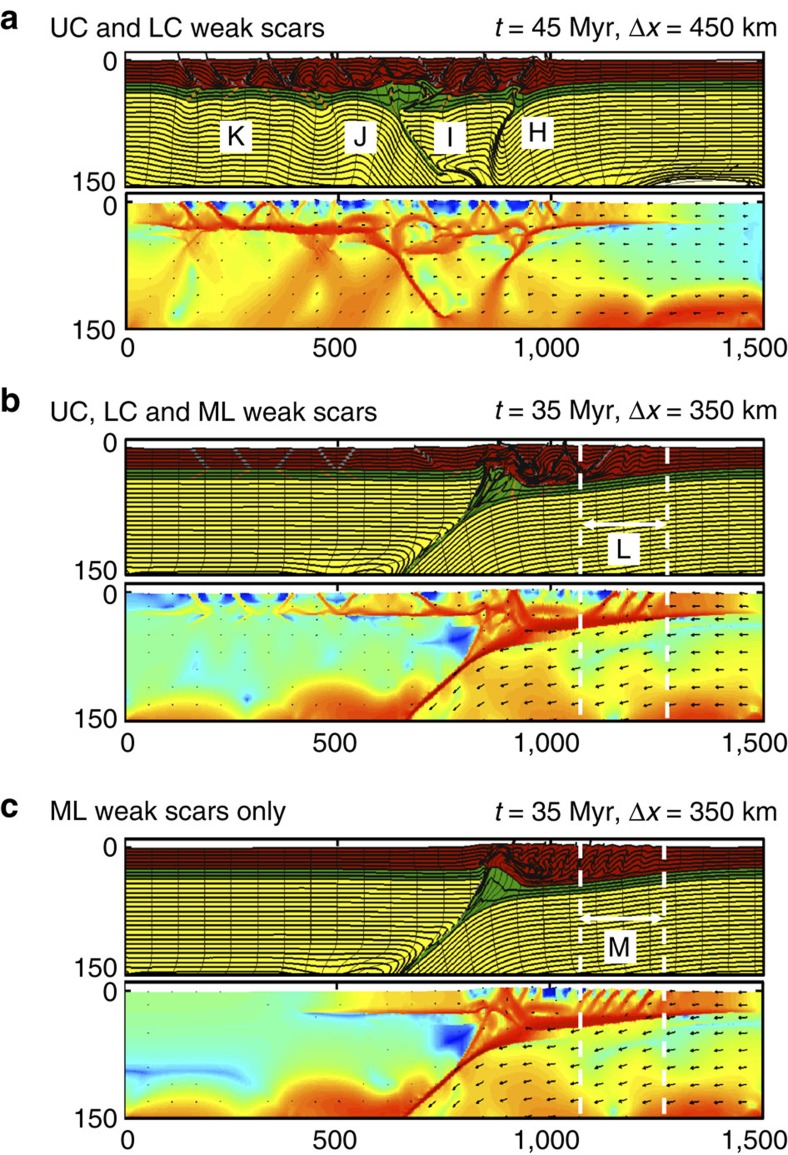
Model results for combinations of weak scars. (**a**) Material deformation (top) and visualization of the second invariant of the deviatoric strain rate tensor (bottom) after 450 km of shortening for a combination of UC and LC weak scars. Markers highlight the order in which deformation occurs. (**b**) As **a**, but including a ML weak scar (configuration given in Fig. 2a). (**c**) Deformation for only ML weak zone. The alphabetical markers (H–K) show the progression of the deformation throughout the full model simulation and track the evolution of tectonics. Where only one alphabetical marker is shown (L and M), the deformation does not move through the full simulation. Dashed lines and markers L and M correspond to the width of deformation after 350 km of shortening.

**Figure 4:**
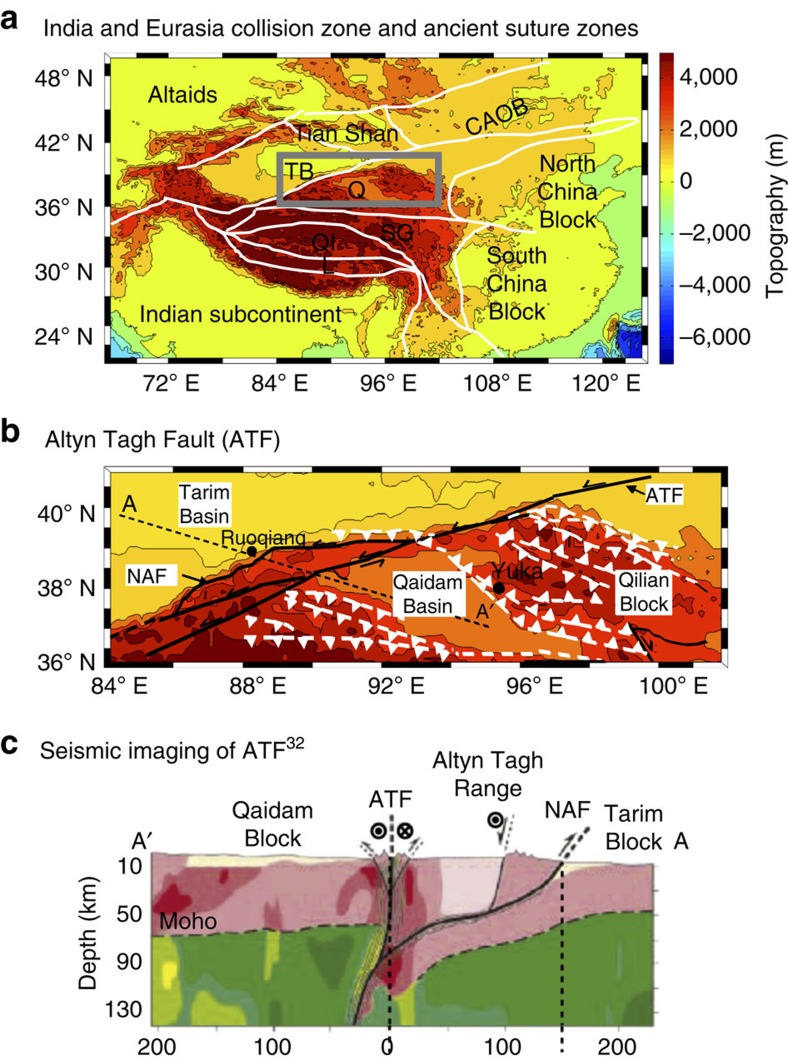
The suture zones of Chinese tectonics and the ATF. (**a**) A topographic map of the different tectonic blocks with paleo-suture zones (white lines) of the India–Eurasia collision zone (suture zones from Watson *et al*.^50^). CAOB, Central Asia Orogenic Belt; L, Lhasa block; Q, Qaidam Basin; QI, Qiantang block; SQ, Songpan–Ganzi complex; TB, Tarim Basin. (**b**) Grey boxed region in **a** showing the ATF with strike-slip faulting denoted in black, with thrust faulting in white^26^. NAF, North Altyn Fault. (**c**) Schematic seismic model of ATF^28^ from Zhang *et al*.^27^. Red and green regions indicate the crust and mantle, respectively. Regions that are more yellow or red in the model are low-velocity zones. Seismic line A to A′ is marked on **b**.

The ability of deep lithospheric heterogeneous structures to exist over long periods in stable continental settings (Fig. 1) allows for a new mechanism for intraplate evolution (following external forcing). If, as an example, the ATF has a long-lasting ML scar from a continental collision that is controlling the crustal evolution, then plate tectonics may indeed display ‘perennial' processes. As such, an increase in intraplate orogenesis would be observed during future (and past) periods of global compression and extension (that is, supercontinent formation and dispersal).

A tenet of the conventional theory of plate tectonics is that crustal deformation is confined to near the boundaries of plates. Our work implies that this remains true for general planetary deformation as ML scars (that can control tectonic evolution) in a continent interior may originate from an ancient plate boundary. In this way, ancient and present-day plate boundaries could be represented together as latent and active boundaries. A global map of perennial plate tectonics (Fig. 1) presents a redefined illustration of tectonic activity and modifies the conventional theory of plate tectonics.

## Methods

### Governing equations

The experiments are modelled using the 2D, thermal–mechanical finite element numerical code SOPALE^43^, which implements an Arbitrary Lagrangian Eulerian method to solve for the deformation of high Prandtl number incompressible viscous–plastic media. The governing hydrodynamic equations for the numerical models include the conservation equations of mass, momentum and internal energy:


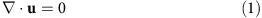



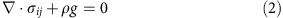



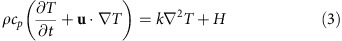


respectively. In the equations above, **u** (m s^−1^), *σ* (Pa), *ρ* (kg m^−3^), *g* (m s^−2^), *c*_*p*_ (J kg^−1^ K^−1^), *T* (K), *k* (W m^−1^ K^−1^), *H* (W m^−3^) and *t* (s) are the velocity, stress tensor, density, gravitational acceleration, specific heat capacity, temperature, thermal conductivity, volumetric rate of internal heat production and time, respectively. The system is completed by an associated linearized equation of state:


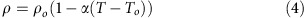


where *α* is the coefficient of thermal expansion, *ρ*_*o*_ is the reference material density and *T*_*o*_ is the reference temperature.

The stress tensor in equation (2) may be divided into the deviatoric stress tensor, *σ'*, and a pressure term,


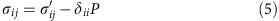


where for an incompressible fluid, *δ* is the Kronecker delta and *P* is the dynamic pressure (which is given as 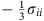). The deviatoric stress is determined at each computational node as the lesser value of a yield stress, *σ*_y_, or viscous stress, *σ*_*ν*_. In the numerical code, the frictional plastic yield stress is given by a pressure-dependent incompressible Drucker–Prager yield criterion


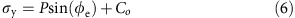


where *φ*_*e*_ is the angle of internal friction and *C*_*o*_ the cohesion. The viscous stress is given by


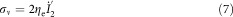


where 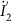 is the second invariant of the deviatoric strain rate tensor, and *η*_e_ the effective viscosity. When the thermally activated power law creep is used, the effective viscosity is given as


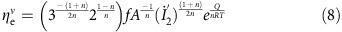


(where *A* is the material constant, *f* is a scaling parameter, *n* is the power law exponent, *Q* is the thermal activation energy, *R* is gas constant and *T* is the temperature).

The Arbitrary Lagrangian Eulerian method calculates creeping flow on a Eulerian grid (resolution here 401 × 217) that is restricted to small vertical dilations (corresponding to the evolution of topography on the free upper surface). The fully deforming Lagrangian grid (resolution 801 × 649) is advected with the computed velocity field and is used to updated the evolving thermal and material properties onto the Eulerian grid (for example, ref. 43).

### Model set-up and material parameters

Supplementary Fig. 1 shows the set-up of the reference case for modelling intraplate deformation. We model the upper and LC, the ML and portion of the sub-lithospheric mantle in 1,500-km width and 600-km depth 2D numerical experiments. The models show convergence in a continental setting, with material properties given in Supplementary Table 1. The geological motivation for this configuration is an old continental lithosphere where ML heterogeneities are found away from plate boundaries (for example, strong crust and ML). The model set-up allows for a heterogeneous lithosphere, with a number of different configurations of weak zones (that is, Supplementary Fig. 3a). As discussed in the manuscript, we model pre-existing zones of weakness by specifying a region with a low angle of internal friction (to mirror the processes of lithospheric damage), and simulate inherited structures in the UC, LC and ML. All weak zones have the same material properties as the layer they occupy, with the specification of a zero internal angle of friction (*φ*_e_). By this method, all faults are equally primed to fail.

An initial (laterally uniform) temperature field (for example, a geotherm) is prescribed to all models. The geotherm has the surface and basal temperature fixed throughout the duration of the model runs (at 20 °C and 1,570 °C, respectively). However, as both the mechanical and thermal calculations are carried out for each time step, the interior temperature field can evolve over space and time, and is not fixed to the preliminary geotherm. Initially, a linear temperature increase of 20–550 °C from the surface to the Moho depth (36 km), with a further linear temperature increase to 1,350 °C at the base of the lithosphere. Radiogenic heat production is applied to the crust, with the majority of internal heat generation coming from the UC (2.1 μW m^−3^) (for example, ref. 44) rather than the LC (0.7 μW m^−3^).

Creeping motion in the model is driven by internal buoyancy forces and an imposed plate motion. In this study, plate motion is modelled by introduced new lithosphere into the domain at the horizontal velocity of *υ*_*c*_=1 cm per year (Supplementary Fig. 1). The left margin of all models is held fixed, while a small outward flux, *υ*_0_, maintains the mass balance of the system (Supplementary Fig. 1).

All calculations were performed on a supercomputer at the SciNet HPC consortium^45^.

Supplementary Fig. 7 shows a recent comparison between two different seismic surveys of the central ATF, specifically a schematic based on the seismic data set used in Wittlinger *et al*.^28^ (Supplementary Fig. 7a) and the more recent study of Zhao *et al*.^29^ (Supplementary Fig. 7b). A ML structure can be seen in both models. Further details of the interpretation of these structures can be found, alongside new magneto-telluric data, in Zhang *et al*.^27^.

### Data availability

The data that support the findings of this study are available from the corresponding author upon request.

## Additional information

**How to cite this article:** Heron, P. J. *et al*. Lasting mantle scars lead to perennial plate tectonics. *Nat. Commun.* 7:11834 doi: 10.1038/ncomms11834 (2016).

## Supplementary Material

Supplementary Information Supplementary Figures 1-7, Supplementary Tables 1 & 2 and Supplementary References.
