# Supplementary material for: Lasting mantle scars lead to perennial plate tectonics
Source: Nat Commun. 2016 Jun 10;7:11834. doi: 10.1038/ncomms11834 (PMC4906409; doi:10.1038/ncomms11834)
Supplement: Supplementary Information — Supplementary Figures 1-7, Supplementary Tables 1 & 2 and Supplementary References. [file ncomms11834-s1.pdf]

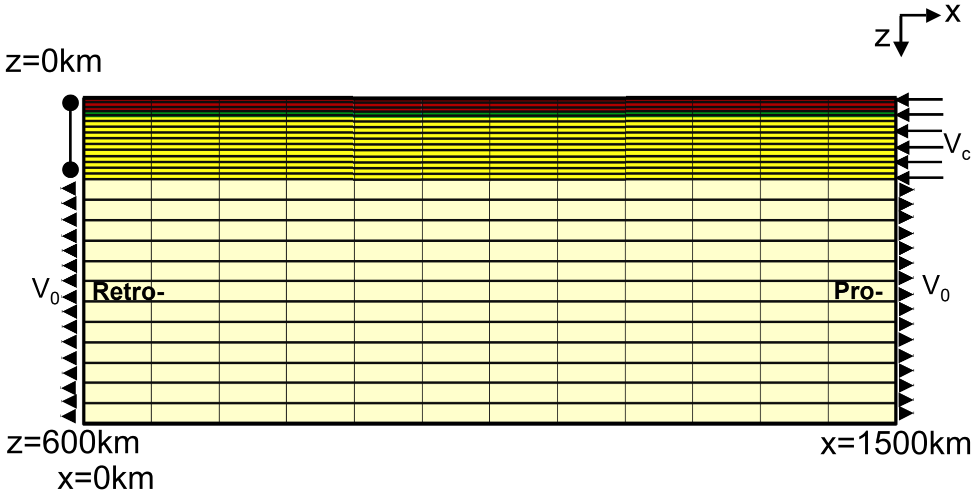

Upper crust thickness: 24km
Lower crust thickness: 12km
Mantle lithosphere thickness: 114km

Lagrangian grid: 801x649
Eulerian grid: 401x217

Supplementary Figure 1: The initial configuration for the numerical experiments, showing the configuration of weak zones. Upper crust (denoted by red and grey colours and has a thickness of 24 km), lower crust (green and blue, thickness 12 km), mantle lithosphere (yellow and white, thickness 114 km), and sub-lithospheric mantle (beige, the bottom 450km). The corresponding physical parameters are given in Supplementary Table 1. Temperature increases linearly throughout the solution domain; surface temperature is 20°C, the Moho temperature is 550°C, the base of the lithosphere is 1350°C, and the base of the model is 1570°C. The Lagrangian and Eulerian grid resolutions are 801$\times$649 and 401$\times$217, respectively. The Lagrangian grid (black mesh) is only partially represented here and shows 17% and 39% of the grid occupies the crust and mantle lithosphere, respectively (the same ratios apply to the Eulerian grid). Continental convergence is incorporated by introducing new lithosphere at the right boundary of box with velocity v_c=1 cm yr^{-1}.

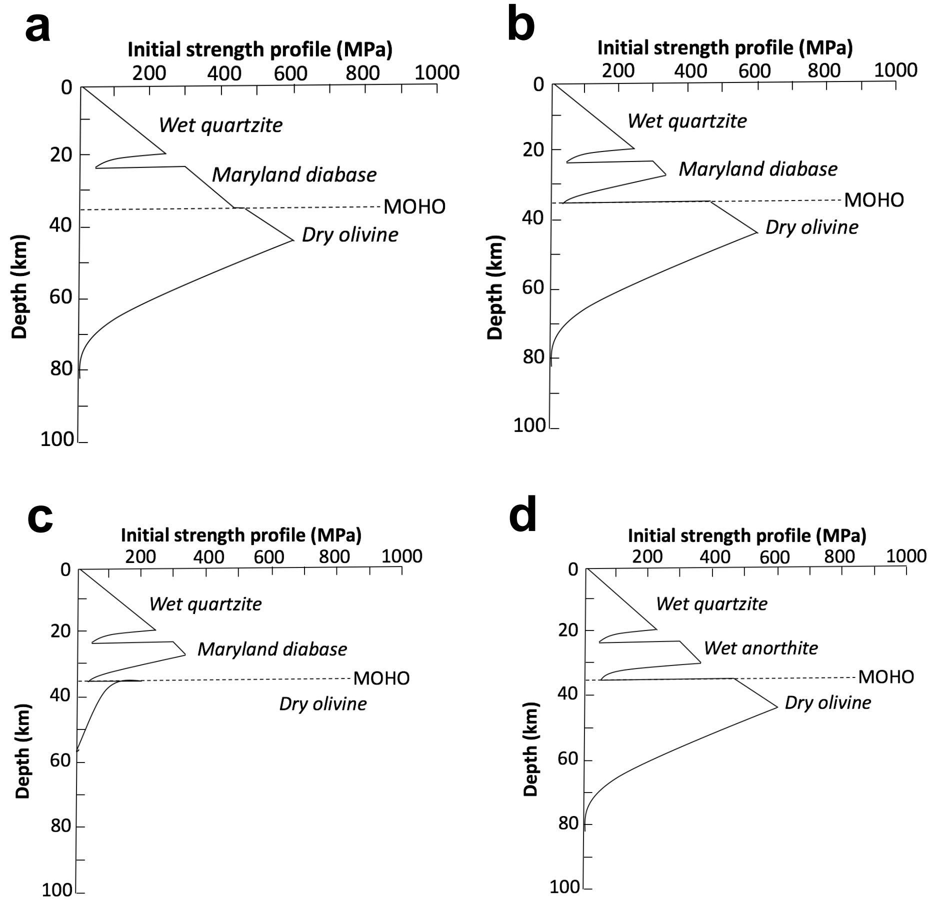

Supplementary Figure 2: Strength envelopes of differential stress versus depth for different rheologies used in the study. The strength of the materials is determined by the minimum of the brittle and viscous strengths. Parameter values are given in Supplementary Table 1 and 2, with the initial geotherm as discussed in Supplementary Table 1 and Supplementary Figure 1. We show a number of different mantle lithosphere scenarios (at a modest representative strain rate of 10^{-15}s^{-1}) to highlight the role of crustal and mantle lithosphere strength in activating tectonics. We present the following rheologic scenarios: **a.** a strong continental crust and mantle lithosphere (as used in the manuscript and Supplementary Table 1); **b.** a weaker lower crust ('jelly sandwich') continental rheology of (a) (Q = 356 kJ mol^{-1} in lower crust (LC)); **c.** a weaker lower crust and mantle lithosphere ('crème brûlée') continental rheology of (b) (Q = 420 kJ mol^{-1} in mantle lithosphere (ML)); **d.** a wet anorthite lower crust (as Supplementary Table 2).

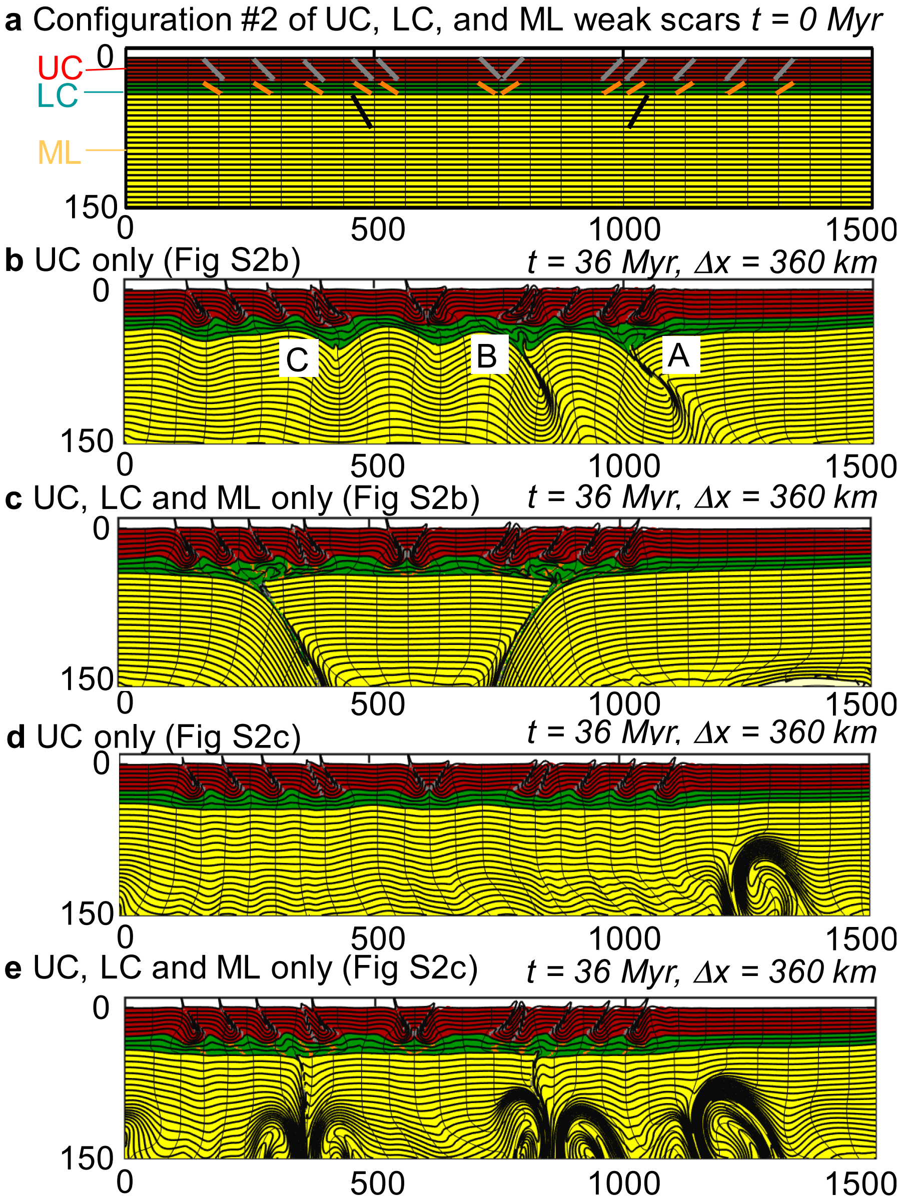

Supplementary Figure 3: Model setup and results from rheological profiles in Supplementary Figure 2b-c. a. Model set-up (red: upper crust (wet quartzite); green: lower crust (Maryland diabase); yellow: mantle lithosphere (dry olivine)). The top 150 km (2x vertical exaggeration) of the 600 km deep model is shown at the initial condition (t = 0 Myr) with the configuration of upper crust (UC), lower crust (LC), and mantle lithosphere (ML) weak scars. The full width of the model is shown. Continental convergence is incorporated by introducing new lithosphere at the right boundary of box with velocity v=1 cm yr^{-1}; **b.** Material deformation for the weaker lower crust ('jelly sandwich') strength profile (Supplementary Figure 2b) with UC only weak zones. The alphabetical markers (A-C) show the progression of the deformation throughout the full model simulation and track the evolution of tectonics; **c.** as b with UC, LC, and ML scars; **d.** Material deformation for weaker lower crust and mantle lithosphere ('crème brûlée') strength profile (Supplementary Figure 2c) with UC only weak zones; **e.** as d with UC, LC, and ML scars.

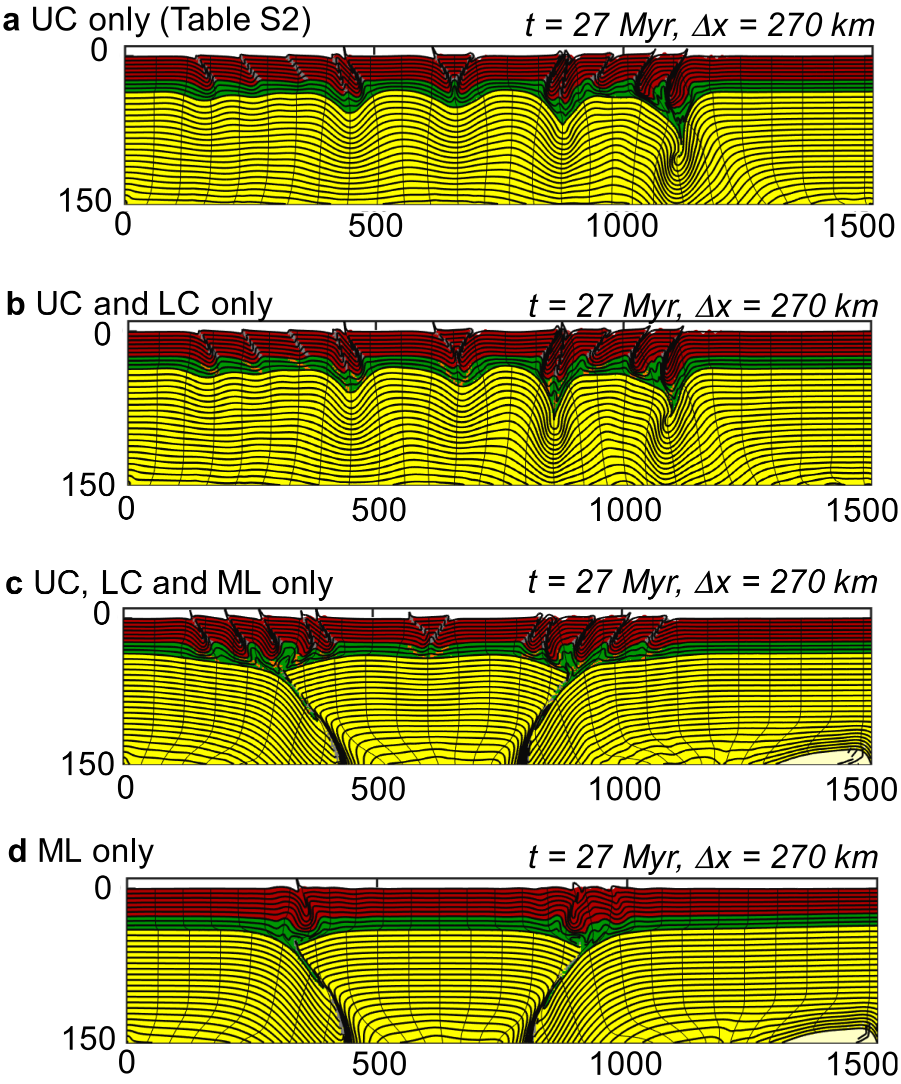

Supplementary Figure 4: Model results from rheological profiles in Supplementary Figure 2d. The material fields for shortening models featuring configuration #2 (Supplementary Figure 3a) with upper crust (UC), lower crust (LC), and mantle lithosphere (ML) flow laws as Supplementary Table 2 and Supplementary Figure 2d. **a.** Upper crust weak scars only; **b.** Upper and lower crust weak scars only; **c.** Upper and lower crust weak scars and two mantle lithosphere scars; **d.** Two mantle lithosphere scars only.

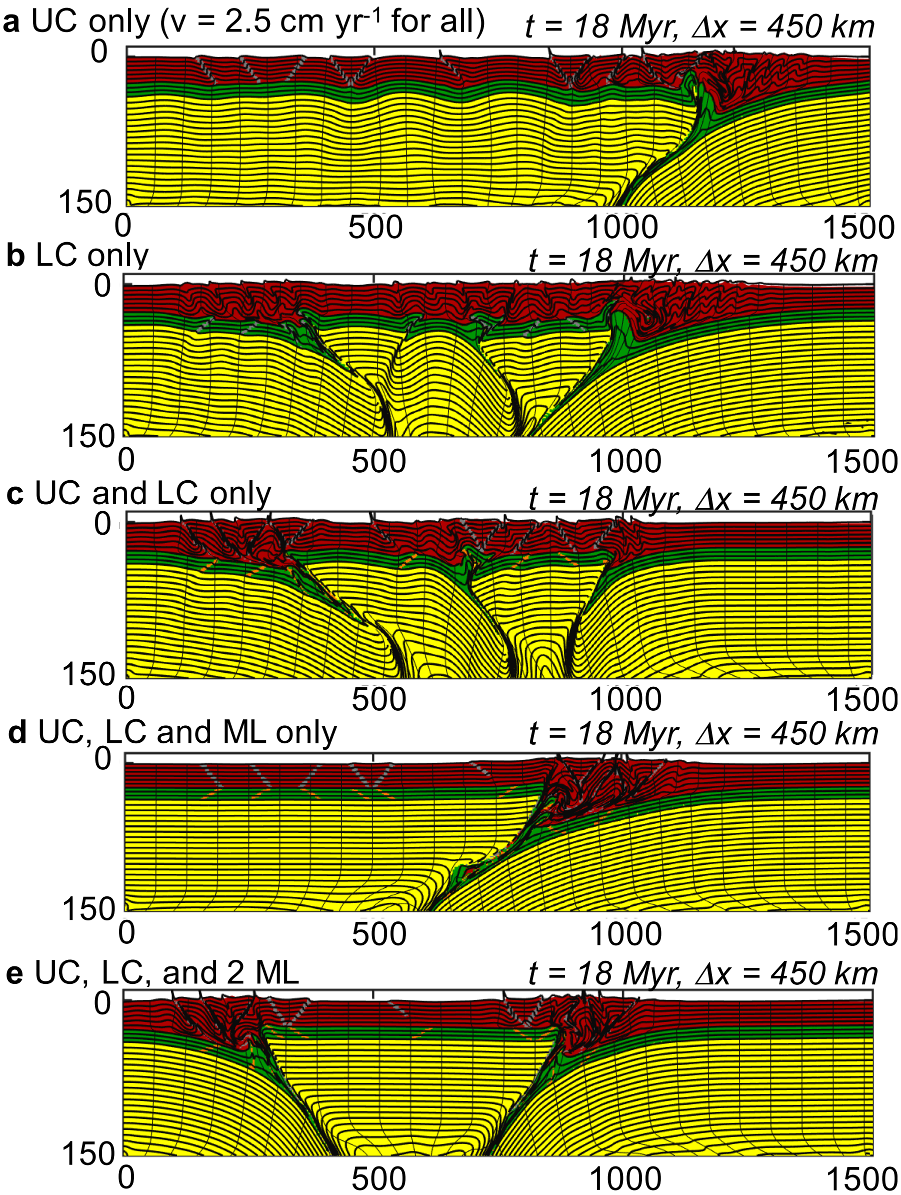

Supplementary Figure 5: Changing the convergence velocity. The material fields for shortening models featuring configuration #2 (Supplementary Figure 3a) with an increased convergence velocity from v = 1 cm yr^{-1} to v = 2.5 cm yr^{-1}. **a.** Upper crust weak scars only. **b.** Lower crust weak scars only. **c.** Upper and lower crust weak scars only. **d.** Upper and lower crust weak scars and one mantle lithosphere scar (as given in by white marker in Figure 2a). **e.** Upper and lower crust weak scars and two mantle lithosphere scars (as given in by black markers in Supplementary Figure 3a).

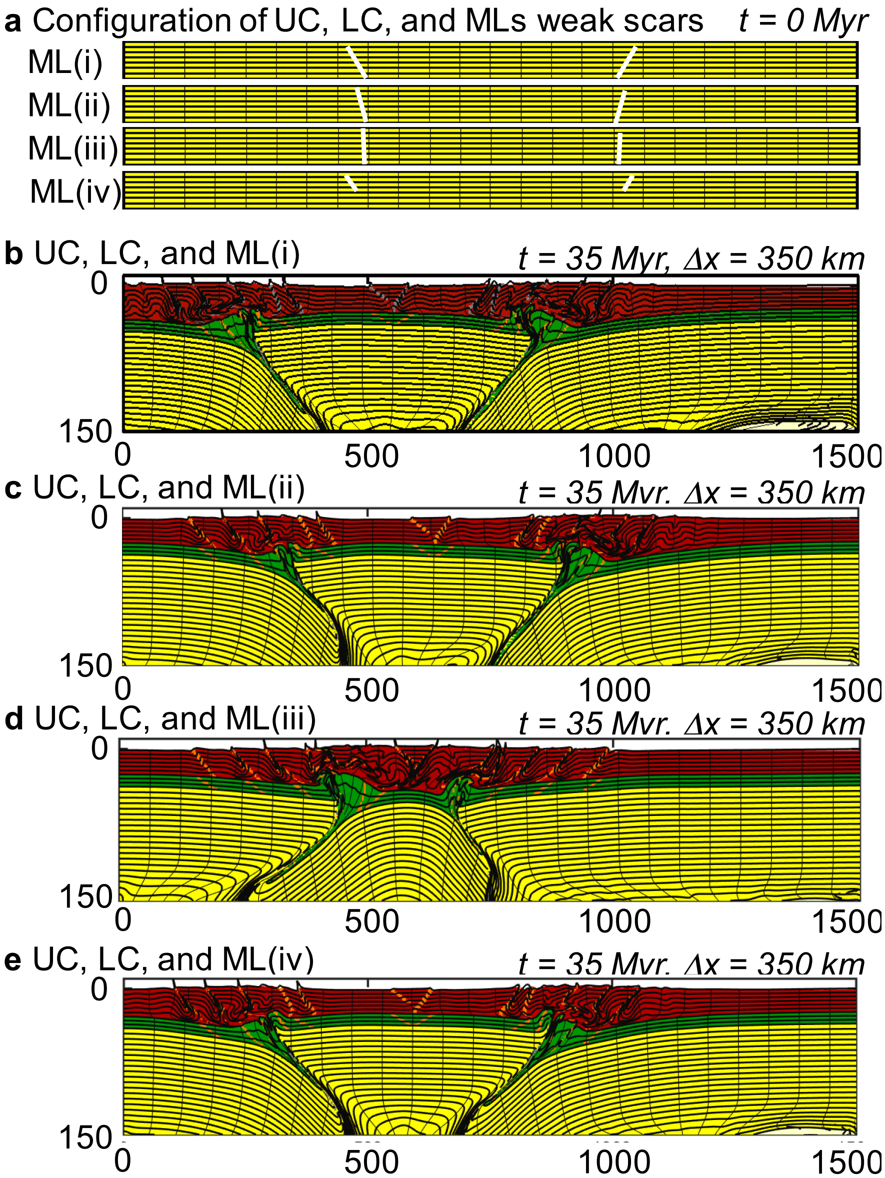

Supplementary Figure 6: Changing the orientation of mantle lithosphere scars. Shortening models are set-up with UC and LC scars as shown in Supplementary Figure 3a. **a.** Configuration of the different orientation of ML scars (for models ML(i - iv)). The deformation pattern after 350 km shortening for ML(i) **b**, ML(ii) **c**, ML(iii) **d**, and ML(iv) **e**.

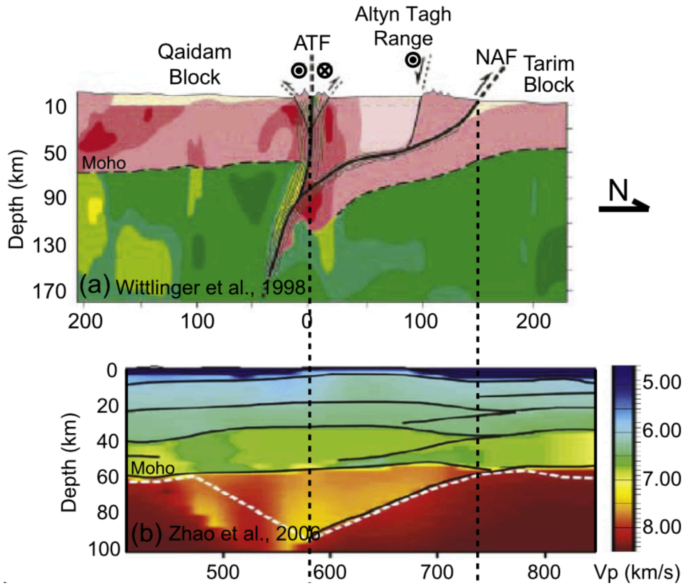

Supplementary Figure 7: Comparison between different geophysical models/datasets across the central Altyn Tagh Fault (ATF). a. Schematic seismic model of Wittlinger et al. [7]. Red and green regions indicate the crust and mantle, respectively. Regions that are more yellow or red in the model are low velocity zones. **b.** Seismic model of Zhao et al. [9]. Black lines are inferred discontinuities from the original paper. Regions above the white dashed line are of relatively high resolution due to dense ray coverage. Horizontal distances in (a) - (b) are using coordinates from the original papers. This figure is taken from Zhang et al. [8]

	C_o	ϕ_2	ϕ_2	$(\dot{I}_2')^{\frac{1}{2}}_1$	$(\dot{I}_2')^{\frac{1}{2}}_2$	A	f	Q	n	Ref	ρ_o	T_0
UC	10^6	15°	2°	0.5	1.5	1.4×10^{-28}	0.3	223	4	1	2700	293
LC	10^6	15°	2°	0.5	1.5	4.4×10^{-29}	0.05	485	4.7	2	2900	293
ML	-	15°	2°	0.5	1.5	8.3×10^{-18}	0.3	535	3.5	3	3250	1609
SM	-	15°	2°	0.5	1.5	8.3×10^{-18}	0.3	535	3.5	3	3250	1609
WZ	*	0	0	*	*	*	*	*		*	*	*

Supplementary Table 1: Rheological parameters used for the continental collisions in the manuscript. Upper crust, lower crust, mantle lithosphere, the sub-lithospheric mantle, and a weak zone are denoted by UC, LC, ML, SM, and WZ, respectively. Symbols are as parameters given in the text (units for C_o, A, Q, ρ_o, and T_0 are Pa, Pa^{-n} s^{-1}, kJ mol^{-1}, K kg m^{-3}, and K). $(\dot{I}_2')^{\frac{1}{2}}$ is accumulated strain. Reference list for Ref: (1) Gleason and Tullis [1]; (2) Ranalli [2], Mackwell et al. [3]; (3) Hirth and Kohlstedt [4], Kawazoe et al. [5]. The flow laws that represent each region are: wet quartzite for UC; Maryland diabase for LC; and dry olivine for ML and SM. For WZ the flow law is governed by the host material (with a prescribed ϕ_e = 0). Physical parameters that remain constant across all regions are α=3$\times$10^{-5}K^{-1}, k=2.25 W m^{-1}K^{-1}, and c_p=750 J kg^{-1} K^{-1}. The upper crust and lower crust have radioactive heat production values of 2.1 μW m^{-3} and 0.7 μW m^{-3}, respectively.

	C_o	ϕ_2	ϕ_2	$(\dot{I}_2')^{\frac{1}{2}}_1$	$(\dot{I}_2')^{\frac{1}{2}}_2$	A	f	Q	n	Ref	ρ_o	T_0
UC	10^6	30°	15°	0.5	1.5	8.6×10^{-28}	0.3	223	4	1	2800	0
LC	10^6	30°	15°	0.5	1.5	1.8×10^{-16}	0.05	356	3	2	2900	0
ML	10^6	30°	15°	0.5	1.5	2.9×10^{-16}	0.3	535	3.5	3	3250	602
SM	10^6	30°	15°	0.5	1.5	2.9×10^{-16}	0.3	535	3.5	3	3300	602
WZ	*	0	0	*	*	*	*	*	*	*	*	*

Supplementary Table 2: A different set of rheological parameters used for the continental collisions. Upper crust, lower crust, mantle lithosphere, the sub-lithospheric mantle, and a weak zone are denoted by UC, LC, ML, SM, and WZ, respectively. * denotes that values are given as the values for which material the weak zone is within. Symbols are as parameters given in the text (units for C_o, A, Q, ρ_o, and T_0 are Pa^{-n}s^{-1}, kJ mol^{-1}, K kg m^{-3}, an K). $(\dot{I}_2')^{\frac{1}{2}}$ is accumulated strain. Reference list for Ref: (1) Gleason and Tullis [1]; (2) Rybacki and Dresen [6]; (3) Hirth and Kohlstedt [4]. The flow laws that represent each region are: wet quartzite for UC; wet anorthite for LC; and dry olivine for ML and SM. For WZ the flow law is governed by the host material (with a prescribed ϕ_e = 0) . Physical parameters that remain constant across all regions are α=3$\times$10^{-5} K^{-1}, k = 2.25 W m^{-1} K^{-1}, and c_p = 750 J kg^{-1} K^{-1}. The upper crust and lower crust have radioactive heat production values of 2.1 μW m^{-3} and 0.7 μW m^{-3}, respectively.

Supplementary References

[1] Gleason G. C. and J. Tullis, A flow law for dislocation creep of quartz aggregates determined with the molten salt cell. *Tectonophysics* **247,** 1-23, (1995).

[2] Ranalli G., Rheology of the lithosphere in space and time. In J.-P. Burg and M. Ford, editors, Orogeny Through Time, **121,** 19-37, London, Geological Society Special Publication, (1997).

[3] Mackwell S.J., Zimmerman, M. E., and Kohlstedt, D. L. High-temperature deformation of dry diabase with application to tectonics on Venus. *Journal of Geophysical Research* **103,** 975-984, (1998).

[4] Hirth G. and Kohlstedt, D. L. Water in the oceanic upper mantle: Implications for rheology, melt extraction and the evolution of the lithosphere. *Earth and Planetary Science Letters* **144,** 93-108, (1996).

[5] Kawazoe T., Karato, S.-I., Otsuka, K., Jing, Z. and Mookherjee, M. Shear deformation of dry polycrystalline olivine under deep upper mantle conditions using a rotational Drickamer apparatus (RDA). *Earth and Planetary Science Letters* **174,** 128-137, (2009).

[6] Rybacki, E., Dresen, G. Dislocation and diffusion creep of synthetic anorthite aggregates. *J. Geophys. Res.* **105,** 26017-26036, (2000).

[7] Wittlinger, G., Tapponnier, P., Poupinet, G., Mei, J., Danian, S., Herquel, G., Masson, F. Tomographic evidence for localized lithospheric shear along the Altyn Tagh Fault. *Science* **282,** 74-76. http://dx.doi.org/10.1126/science.282.5386.74, (1998).

[8] Zhang, L., Unsworth, M., Jin, S., Wei, W., Ye, G., Jones, A. G., Jing, J., Dong, H., Xie. C., Le Pape, F., Vozar., J. Structure of the Central Altyn Tagh Fault revealed by magnetotelluric data: New insights into the structure of the northern margin of the India-Asia collision. *Earth Planet. Sci. Lett.* **415,** 67-79, (2015).

[9] Zhao, J., Mooney, W.D., Zhang, X., Li, Z., Jin, Z., Okaya, N. Crustal structure across the Altyn Tagh Range at the northern margin of the Tibetan Plateau and tectonic implications. *Earth Planet. Sci. Lett.* **241,** 804-814. http://dx.doi.org/ 10.1016/j.epsl.2005.11.003, (2006).
